# A polymorph of terephthalaldehyde

**DOI:** 10.1107/S1600536808022381

**Published:** 2008-07-23

**Authors:** Lei Teng, Zhiguo Wang

**Affiliations:** a School of Chemical and Materials Engineering, Huangshi Institute of Technology, Huangshi 435003, People’s Republic of China

## Abstract

A new ortho­rhom­bic polymorph of terephthalaldehyde, C_8_H_6_O_2_, with a melting point of 372 K, has been obtained by recrystallization from ethanol. At room temperature, the crystals transform into the well known monoclinic form, with a melting point of 389 K. The crystal structure of the monoclinic form involves C—H⋯O hydrogen bonds, but no such bonds are observed in the orthorhombic form. The molecule is planar.

## Related literature

For the structure of the monoclinic polymorph, see: Britton (1998 ▶).
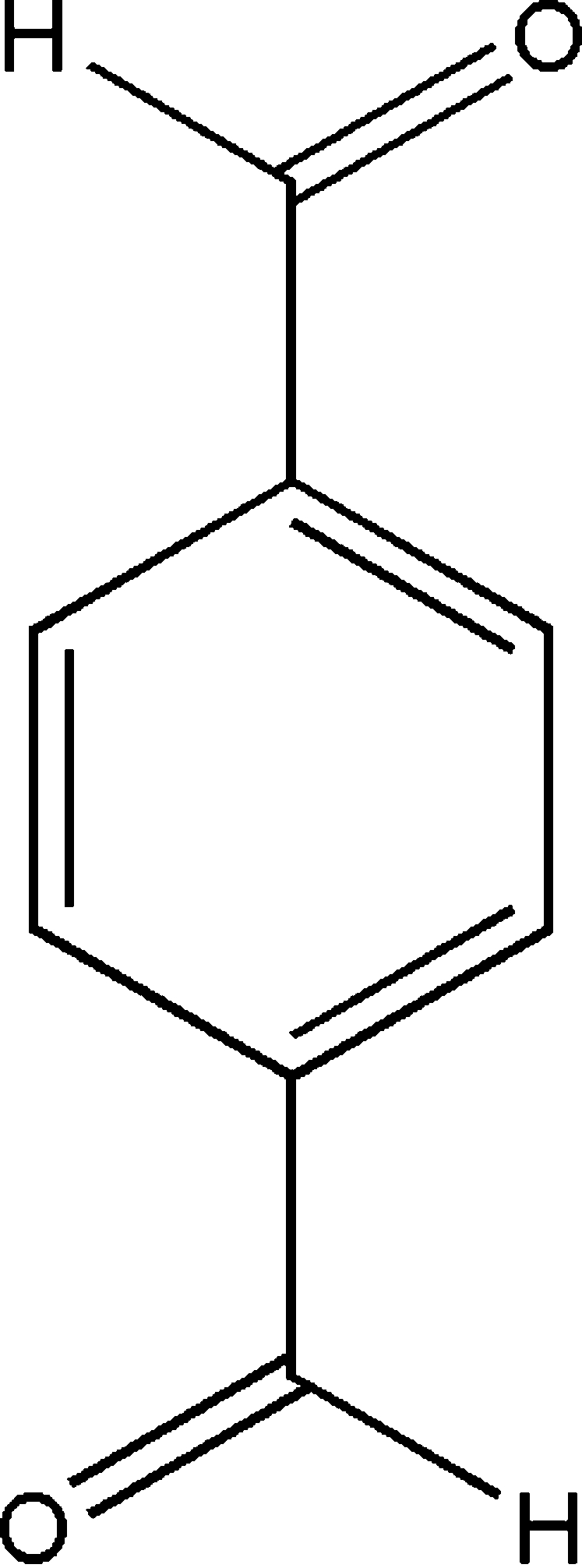


## Experimental

### 

#### Crystal data

C_8_H_6_O_2_
*M* *_r_* = 134.13
Orthorhombic, 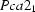

*a* = 12.8811 (5) Å
*b* = 3.8933 (3) Å
*c* = 13.3202 (9) Å
*V* = 668.01 (7) Å^3^
*Z* = 4
Mo *K*α radiation
μ = 0.10 mm^−1^
*T* = 295 (2) K
0.20 × 0.10 × 0.10 mm

#### Data collection

Bruker SMART 4K CCD area-detector diffractometer
Absorption correction: none
3959 measured reflections
653 independent reflections
563 reflections with *I* > 2σ(*I*)
*R*_int_ = 0.030

#### Refinement

*R*[*F* ^2^ > 2σ(*F* ^2^)] = 0.042
*wR*(*F* ^2^) = 0.112
*S* = 1.04
653 reflections
91 parameters
1 restraint
H-atom parameters constrained
Δρ_max_ = 0.16 e Å^−3^
Δρ_min_ = −0.10 e Å^−3^

### 

Data collection: *SMART* (Bruker, 2001 ▶); cell refinement: *SAINT* (Bruker, 2001 ▶); data reduction: *SAINT*; program(s) used to solve structure: *SHELXS97* (Sheldrick, 2008 ▶); program(s) used to refine structure: *SHELXL97* (Sheldrick, 2008 ▶); molecular graphics: *SHELXTL* (Sheldrick, 2008 ▶); software used to prepare material for publication: *SHELXTL*.

## Supplementary Material

Crystal structure: contains datablocks I, global. DOI: 10.1107/S1600536808022381/bq2093sup1.cif

Structure factors: contains datablocks I. DOI: 10.1107/S1600536808022381/bq2093Isup2.hkl

Additional supplementary materials:  crystallographic information; 3D view; checkCIF report

## Figures and Tables

**Fig. 1.**
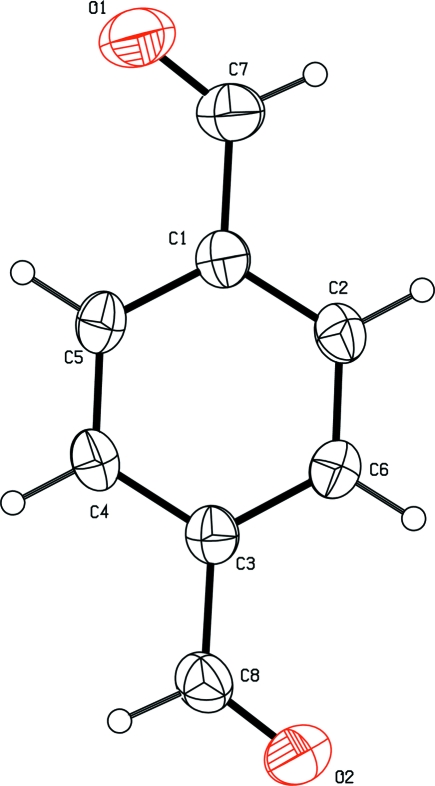
The molecular structure of (I) with 50% probability displacement ellipsoids (arbitrary spheres for H atoms).

**Fig. 2.**
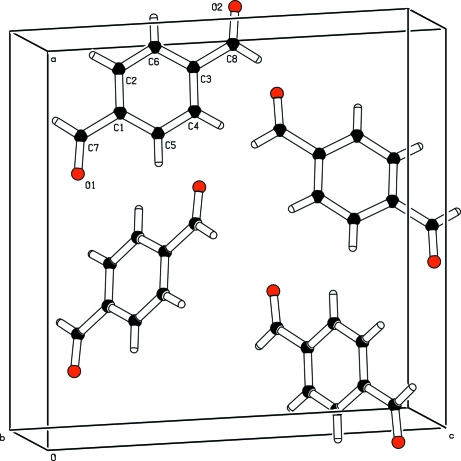
The packing of the molecules, viewed down the b axis.

## supplementary crystallographic information

### Comment

Terephthalaldehyde, (I), is a simple aromatic compound used in organic synthesis. It is known as a white substance, slightly soluble in water, with a melting point of 389 K. To the best of our knowledge, the only packing information of (I) in the solid state comes from the work of Britton (Britton, 1998), who investigated the intermolecular C—H···O arrangement in the structure of terephthalaldehyde and the crystals suitable for diffraction were obtained by recrystallization from an acetone/ethyl ether mixture.

A new polymorphic form of (I) has been obtained serendipitously during cocrystallization of pyridine and terephthalaldehyde from ethanol (Fig. 1). Flat needles which precipitated first from the solution had a melting point of 372 K and were identified as a new polymorphic form of (I) by X-ray crystallography. At room temperature, the crystals transform into the well known monoclinic form, with a melting point of 389 K. The crystal structure of the monoclinic form involves C—H···O hydrogen bonds but no such bonds are observed in the orthorhombic form (Fig. 2).

### Experimental

The title compound was purchased from Aldrich.

### Refinement

All H atoms were initially located in a difference Fourier map and then included with constrained bond lengths and isotropic displacement parameters: C—H=0.93Å and *U*_iso_(H)=1.2*U*_eq_(C) for H atoms.

### Crystal data

**Table:**

C_8_H_6_O_2_	*F*_000_ = 280
*M**_r_* = 134.13	*D*_x_ = 1.334 Mg m^−^^3^
Orthorhombic, *P**c**a*2_1_	Mo *K*α radiation λ = 0.71073 Å
Hall symbol: P 2c -2ac	Cell parameters from 1070 reflections
*a* = 12.8811 (5) Å	θ = 3.1–23.5º
*b* = 3.8933 (3) Å	µ = 0.10 mm^−^^1^
*c* = 13.3202 (9) Å	*T* = 295 (2) K
*V* = 668.01 (7) Å^3^	Block, colorless
*Z* = 4	0.20 × 0.10 × 0.10 mm

### Data collection

**Table:**

Bruker SMART 4K CCD area-detector diffractometer	563 reflections with *I* > 2σ(*I*)
Radiation source: fine-focus sealed tube	*R*_int_ = 0.030
Monochromator: graphite	θ_max_ = 25.5º
*T* = 295(2) K	θ_min_ = 3.1º
φ and ω scans	*h* = −15→12
Absorption correction: none	*k* = −4→4
3959 measured reflections	*l* = −16→15
653 independent reflections	

### Refinement

**Table:**

Refinement on *F*^2^	Secondary atom site location: difference Fourier map
Least-squares matrix: full	Hydrogen site location: inferred from neighbouring sites
*R*[*F*^2^ > 2σ(*F*^2^)] = 0.042	H-atom parameters constrained
*wR*(*F*^2^) = 0.112	*w* = 1/[σ^2^(*F*_o_^2^) + (0.0755*P*)^2^ + 0.0133*P*] where *P* = (*F*_o_^2^ + 2*F*_c_^2^)/3
*S* = 1.04	(Δ/σ)_max_ = 0.014
653 reflections	Δρ_max_ = 0.16 e Å^−^^3^
91 parameters	Δρ_min_ = −0.10 e Å^−^^3^
1 restraint	Extinction correction: none
Primary atom site location: structure-invariant direct methods	

### Special details

**Table:**

Geometry. All e.s.d.'s (except the e.s.d. in the dihedral angle between two l.s. planes) are estimated using the full covariance matrix. The cell e.s.d.'s are taken into account individually in the estimation of e.s.d.'s in distances, angles and torsion angles; correlations between e.s.d.'s in cell parameters are only used when they are defined by crystal symmetry. An approximate (isotropic) treatment of cell e.s.d.'s is used for estimating e.s.d.'s involving l.s. planes.
Refinement. Refinement of *F*^2^ against ALL reflections. The weighted *R*-factor *wR* and goodness of fit *S* are based on *F*^2^, conventional *R*-factors *R* are based on *F*, with *F* set to zero for negative *F*^2^. The threshold expression of *F*^2^ > σ(*F*^2^) is used only for calculating *R*-factors(gt) *etc*. and is not relevant to the choice of reflections for refinement. *R*-factors based on *F*^2^ are statistically about twice as large as those based on *F*, and *R*- factors based on ALL data will be even larger.

### Fractional atomic coordinates and isotropic or equivalent isotropic displacement parameters (Å^2^)

**Table:**

	*x*	*y*	*z*	*U*_iso_*/*U*_eq_	
C1	0.8164 (2)	0.3463 (7)	0.2145 (2)	0.0509 (7)	
C2	0.9236 (2)	0.3850 (7)	0.2147 (3)	0.0545 (7)	
H2	0.9566	0.4847	0.1599	0.065*	
C3	0.9315 (2)	0.1279 (7)	0.3776 (2)	0.0515 (7)	
C4	0.8239 (2)	0.0894 (7)	0.3770 (3)	0.0547 (7)	
H4	0.7907	−0.0106	0.4316	0.066*	
C5	0.7668 (2)	0.1993 (6)	0.2955 (3)	0.0543 (7)	
H5	0.6950	0.1744	0.2953	0.065*	
C6	0.9805 (2)	0.2776 (7)	0.2951 (3)	0.0547 (6)	
H6	1.0523	0.3040	0.2949	0.066*	
C7	0.7563 (4)	0.4635 (8)	0.1269 (4)	0.0707 (10)	
H7	0.7934	0.5547	0.0733	0.085*	
C8	0.9916 (3)	0.0084 (8)	0.4657 (4)	0.0627 (9)	
H8	0.9550	−0.0874	0.5190	0.075*	
O1	0.6637 (2)	0.4514 (7)	0.1187 (3)	0.0930 (10)	
O2	1.0834 (2)	0.0266 (7)	0.4731 (4)	0.0876 (9)	

### Atomic displacement parameters (Å^2^)

**Table:**

	*U*^11^	*U*^22^	*U*^33^	*U*^12^	*U*^13^	*U*^23^
C1	0.0535 (16)	0.0494 (14)	0.0498 (17)	0.0041 (11)	0.0057 (14)	−0.0068 (13)
C2	0.0564 (18)	0.0551 (15)	0.0520 (17)	0.0022 (13)	0.0123 (15)	0.0015 (15)
C3	0.0547 (16)	0.0509 (14)	0.0490 (17)	−0.0008 (12)	0.0064 (15)	−0.0078 (13)
C4	0.0593 (19)	0.0573 (14)	0.0476 (17)	−0.0067 (14)	0.0140 (16)	0.0002 (14)
C5	0.0463 (13)	0.0570 (14)	0.0595 (15)	−0.0016 (12)	0.0102 (17)	−0.0083 (14)
C6	0.0448 (13)	0.0582 (14)	0.0610 (15)	−0.0003 (12)	0.0043 (18)	−0.0008 (14)
C7	0.071 (2)	0.077 (2)	0.063 (3)	0.0121 (16)	−0.006 (2)	−0.0016 (18)
C8	0.067 (2)	0.0704 (19)	0.0508 (19)	0.0030 (13)	0.009 (2)	0.0003 (14)
O1	0.075 (2)	0.129 (2)	0.075 (2)	0.0162 (14)	−0.0158 (19)	−0.0026 (18)
O2	0.0617 (17)	0.125 (2)	0.0761 (19)	0.0064 (12)	−0.0077 (16)	0.0074 (15)

### Geometric parameters (Å, °)

**Table:**

C1—C5	1.378 (4)	C4—C5	1.379 (5)
C1—C2	1.389 (4)	C4—H4	0.9300
C1—C7	1.473 (6)	C5—H5	0.9300
C2—C6	1.363 (5)	C6—H6	0.9300
C2—H2	0.9300	C7—O1	1.199 (5)
C3—C4	1.393 (4)	C7—H7	0.9300
C3—C6	1.395 (4)	C8—O2	1.188 (4)
C3—C8	1.482 (6)	C8—H8	0.9300
			
C5—C1—C2	120.2 (3)	C1—C5—C4	119.9 (3)
C5—C1—C7	120.4 (3)	C1—C5—H5	120.1
C2—C1—C7	119.4 (3)	C4—C5—H5	120.1
C6—C2—C1	120.2 (3)	C2—C6—C3	120.2 (2)
C6—C2—H2	119.9	C2—C6—H6	119.9
C1—C2—H2	119.9	C3—C6—H6	119.9
C4—C3—C6	119.4 (3)	O1—C7—C1	125.6 (5)
C4—C3—C8	119.4 (3)	O1—C7—H7	117.2
C6—C3—C8	121.2 (3)	C1—C7—H7	117.2
C5—C4—C3	120.1 (3)	O2—C8—C3	124.5 (4)
C5—C4—H4	120.0	O2—C8—H8	117.7
C3—C4—H4	120.0	C3—C8—H8	117.7
			
C5—C1—C2—C6	0.1 (4)	C1—C2—C6—C3	0.0 (4)
C7—C1—C2—C6	−179.9 (3)	C4—C3—C6—C2	0.0 (4)
C6—C3—C4—C5	−0.2 (4)	C8—C3—C6—C2	179.9 (3)
C8—C3—C4—C5	180.0 (3)	C5—C1—C7—O1	1.8 (5)
C2—C1—C5—C4	−0.2 (4)	C2—C1—C7—O1	−178.2 (3)
C7—C1—C5—C4	179.7 (3)	C4—C3—C8—O2	179.2 (3)
C3—C4—C5—C1	0.2 (4)	C6—C3—C8—O2	−0.6 (5)
